# The recurrent chromosomal translocation t(12;18) (q14~15;q12~21) causes the fusion gene *HMGA2-SETBP1* and *HMGA2* expression in lipoma and osteochondrolipoma

**DOI:** 10.3892/ijo.2015.3099

**Published:** 2015-07-21

**Authors:** IOANNIS PANAGOPOULOS, LUDMILA GORUNOVA, BODIL BJERKEHAGEN, INGVILD LOBMAIER, SVERRE HEIM

**Affiliations:** 1 Section for Cancer Cytogenetics, Institute for Cancer Genetics and Informatics, The Norwegian Radium Hospital, Oslo University Hospital, Oslo, Norway; 2 Centre for Cancer Biomedicine, Faculty of Medicine, University of Oslo, Oslo, Norway; 3 Department of Pathology, The Norwegian Radium Hospital, Oslo University Hospital, Oslo, Norway; 4 Faculty of Medicine, University of Oslo, Oslo, Norway

**Keywords:** lipoma, osteochondrolipoma, t(12;18)(q14~15;q12~21), recurrent chromosomal translocation, *HMGA2-SETBP1*, *HMGA2* expression

## Abstract

Lipomas are the most common soft tissue tumors in adults. They often carry chromosome aberrations involving 12q13~15 leading to rearrangements of the *HMGA2* gene in 12q14.3, with breakpoints occurring within or outside of the gene. Here, we present eleven lipomas and one osteochondrolipoma with a novel recurrent chromosome aberration, t(12;18) (q14~15;q12~21). Molecular studies on eight of the tumors showed that full-length *HMGA2* transcript was expressed in three and a chimeric *HMGA2* transcript in five of them. In three lipomas and in the osteochondrolipoma, exons 1–3 of *HMGA2* were fused to a sequence of *SETBP1* on 18q12.3 or an intragenic sequence from 18q12.3 circa 10 kbp distal to *SETBP1*. In another lipoma, exons 1–4 of *HMGA2* were fused to an intronic sequence of *GRIP1* which maps to chromosome band 12q14.3, distal to *HMGA2*. The ensuing *HMGA2* fusion transcripts code for putative proteins which contain amino acid residues of HMGA2 corresponding to exons 1–3 (or exons 1–4 in one case) followed by amino acid residues corresponding to the fused sequences. Thus, the pattern is similar to the rearrangements of *HMGA2* found in other lipomas, i.e., disruption of the *HMGA2* locus leaves intact exons 1–3 which encode the AT-hooks domains and separates them from the 3′-terminal part of the gene. The fact that the examined osteochondrolipoma had a t(12;18) and a *HMGA2-SETBP1* fusion identical to the findings in the much more common ordinary lipomas, underscores the close developmental relationship between the two tumor types.

## Introduction

Lipomas are benign tumors composed of mature fat cells (1). It is the most common soft tissue tumor in adults with peak incidence between 40–60 years. They may appear at any site but with a broad distinction between subcutaneous (superficial) and deep-seated lesions. Although most lipomas are easily diagnosed, those occurring deep down (e.g., intramuscular lipoma, perineural lipoma) may be confused with liposarcomas. Though mitoses are rarely seen in histologic sections of lipomas, karyotypes can easily be obtained from short-term cultured tumor cells. In 1986, the first characteristic acquired chromosome aberration of lipomas was described, the translocation t(3;12)(q27~28;q13~15) (2,3). Since then, according to the Mitelman Database of Chromosome Aberrations and Gene Fusions in Cancer (http://cgap.nci.nih.gov/Chromosomes/Mitelman database updated on February 13, 2015), 476 lipomas with chromosome aberrations have been reported, with involvement of chromosome bands 12q13~15 being seen in more than 300 of them. Recombination may take place with a wide variety of partners, but the by far most common is the translocation t(3;12)(q27~28;q14~15) which has been reported in 53 of the aberrant karyotypes registered in the Mitelman Database. Other recurrently involved chromosome segments found recombined with 12q13~15 in lipomas are 1p36 and 1p32~34 (each found in 29 cases), 2p22~24 (in 6 cases), 2q35~37 (10 cases), 5q33 (16 cases), 9p21~22 (7 cases) and 12p11~13 and 13q12~14 (8 cases each). The chromosome rearrangements often target the *HMGA2* gene in 12q14.3 with breakpoints occurring both within and outside the locus; the essential outcome appears to be deregulation of *HMGA2* with truncation of the gene as the critical event (4 and refs. therein). The most frequent translocation, t(3;12)(q27~28;q13~15), generates an *HMGA2-LPP* fusion gene coding for a transcription factor containing the AT-hook domain of *HMGA2* and C-terminal LIM domains of LPP (5,6). *HMGA2* has also been reported to form fusion genes with *PPAP2* (at 1p32), *ACKR3* (at 2q37), *EBF1* (at 5q33), *NFIB* (at 9p22) and *LHFP* (at 13q12) (7–11). In the present study, we describe a novel recurrent chromosome translocation, t(12;18)(q14~15;q12~21) and its molecular consequences in 12 benign fat cell tumors.

## Materials and methods

### Ethics statement

The study was approved by the regional ethics committee (Regional komité for medisinsk forskning-setikk Sør-Øst, Norge, http://helseforskning.etikkom.no) and written informed consent was obtained from the patients.

### Patients

Table I shows the patients' gender, age, diagnosis and the location of the tumor. All tumors were surgically removed.

### Chromosome banding analysis

Samples from the operation specimens were mechanically and enzymatically disaggregated and short-term cultured as described elsewhere (12). The cultures were harvested and the chromosomes G-banded using Wright stain. The subsequent cytogenetic analysis and karyotype description followed the recommendations of the ISCN (13).

### Total RNA isolation and cDNA synthesis

Tumor tissue adjacent to that used for cytogenetic analysis and histologic examination had been frozen and stored at −80°C from eight tumors (cases 1–8). Total RNA was extracted using miRNeasy kit, TissueLyser II homogenizer and Qiacube according to the manufacturer's recommendations (Qiagen Nordic, Stockholm, Sweden). For cDNA synthesis, 400–500 ng of total RNA were reverse-transcribed in a 20 μl reaction volume using iScript Advanced cDNA Synthesis kit for RT-qPCR according to the manufacturer's instructions (Bio-Rad Laboratories, Oslo, Norway). cDNA equivalent to 10 ng/μl of total RNA was used as template in subsequent real-time PCR assays. The Human Universal Reference Total RNA was used as control (Clontech Laboratories, Takara Bio Group, Saint-Germainen-Laye, France). According to the company's information, it is a mixture of total RNAs from a collection of adult human tissues, chosen to represent a broad range of expressed genes. Both male and female donors are represented.

### Real-time PCR

Real-time PCR was carried out to determine the expression level of *HMGA2*. The TaqMan gene expression assays (Applied Biosystems, Foster City, CA, USA) Hs00171569_m1 (*HMGA2* exons 1–2) and Hs00971725_m1 (HMGA2 exons 4–5) were used. The *ACTB* gene, assay Hs99999903_m1, was used as endogenous control. The 20 μl reaction volume contained 1× TaqMan Universal Master Mix II with UNG, lX of the 20× TaqMan Gene Expression Mix, and 1 μl cDNA (10 ng equivalent of RNA). Four replicates of each sample and the endogenous control were performed. Real-time PCR was run on a CFX96 Touch™ Real-Time PCR Detection System (Bio-Rad Laboratories). The thermal cycling included an initial step at 50°C for 2 min, followed by 10 min at 95°C, 40 cycles of 15 sec at 95°C and 1 min at 60°C. The data were analyzed using Bio-Rad CFX Manager software (Bio-Rad Laboratories).

### 3′-Rapid amplification cDNA ends (3′-RACE)

For 3′-RACE, 100 ng of total RNA were reverse-transcribed in a 20 μl reaction volume with the A3RNV-RACE primer (5′-ATC GTT GAG ACT CGT ACC AGC AGA GTC ACG AGA GAG ACT ACA CGG TAC TGG TTT TTT TTT TTT TTT-3′) using iScript Select cDNA Synthesis kit according to the manufacturer's instructions (Bio-Rad Laboratories). One microliter was used as template and amplified using the outer primer combination HMGA2-846F1 (5′-CCA CTT CAG CCC AGG GAC AAC CT-3′) and A3R-1New (5′-TCG TTG AGA CTC GTA CCA GCA GAG TCA C-3′). One microliter of the amplified products was used as template in nested PCR with the primers HMGA2-982F1 (5′-CAA GAG TCC CTC TAA AGC AGC TCA-3′) and A3R-3 (5′-CGA GAG AGA CTA CAC GGT ACT GGT-3′). For both PCRs, the 25 μl reaction volume contained 12.5 μl of Premix Taq (Takara Bio), template, and 0.4 μM of each of the forward and reverse primers. PCR cycling consisted of an initial step of denaturation at 94°C for 30 sec followed by 35 cycles of 7 sec at 98°C, 30 sec at 55°C, 90 sec at 72°C, and a final extension for 5 min at 72°C.

A total of 3 μl of the PCR products were stained with GelRed (Biotium, Hayward, CA, USA), analyzed by electrophoresis through 1.0% agarose gel and photographed. The rest of the amplified fragments were purified using the Thermo Scientific GeneJET PCR purification kit (Thermo Fisher Scientific, Oslo, Norway) and direct sequencing was performed using the light run sequencing service of GATC Biotech (http://www.gatc-biotech.com/en/sanger-services/lightrun-sequencing.html). The BLAST (http://blast.ncbi.nlm.nih.gov/Blast.cgi) and BLAT (http://genome.ucsc.edu/cgi-bin/hgBlat) programs were used for computer analysis of sequence data.

### Reverse transcriptase PCR (RT-PCR)

To verify the results obtained by 3′-RACE, i.e., the presence of *HMGA2*-chimeric transcript (see below), PCRs were performed using the forward primer HMGA2-846F1 and the reverse primer SETBP1-5390R1 (5′-GCA GCG TGA GGT CAG GAG AGT GC-3′) for cases 4 and 7. The primers HMGA2-846F1 and SETBP1-5325F1 (5′-GGC GCT TCA GTA CGG CTG GAT C-3′) were used for case 8. For case 3, the primer HMGA2-846F1 and the reverse primer 18q21-Rev1 (5′-GCA TTG GCA GTC CCC TTG CAT T -3′) were used. For case 5, the primer HMGA2-846F1 and the reverse primer GRIP-intrR1 (5′-TTA AGG TGT GGC CTT TAG GCG TGA C-3′) were used. The 25 μl PCR volumes contained 12.5 μl of Premix Taq (Takara Bio), 1 μl of diluted cDNA (10 ng equivalent of RNA), and 0.4 μM of each of the forward and reverse primers. The PCRs were run on a C-1000 Thermal cycler (Bio-Rad Laboratories). The PCR conditions were: an initial denaturation at 94°C for 30 sec followed by 35 cycles of 7 sec at 98°C, 120 sec at 68°C, and a final extension for 5 min at 68°C.

## Results

### Pathology and cytogenetics

**Figure 1:**
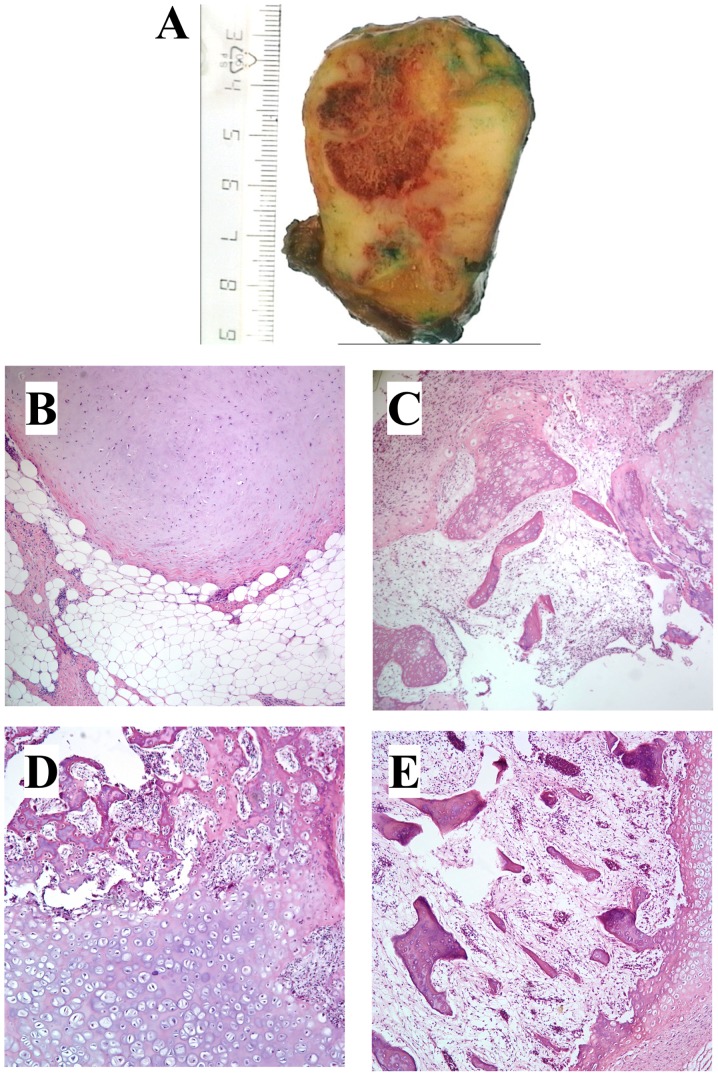
Macroscopical and microscopical examination (H&E stained slides) of the osteochondrolipoma. (A) Cut surface of the osteochondrolipoma with macroscopically visible fatty tissue, cartilage and bone. (B) Mature cartilage nodule within fatty tissue. (C) Mature cartilage with bone formation and fatty tissue with lymphoid infiltration. (D) Mature cartilage and bone. (E) Mature cartilage and bone with bone marrow elements.

All tumors were ordinary lipomas except case 8, which was an osteochondrolipoma (Table I and Fig. 1), a rare variant of lipoma with metaplastic components of cartilage and bone within the fatty tissue. In this tumor, microscopic examination showed lobules of mature fat separated by strands of fibrous tissue and areas with hyaline cartilage. Other areas showed bony trabeculae with bone marrow between them (Fig. 1). There was no cellular atypia. Reactive changes with prominent lymphoid infiltrates in the bone marrow were seen, and in the fatty tissue and fibrous septa there were mature lymphocytes and some plasma cells (Fig. 1).

**Figure 2:**
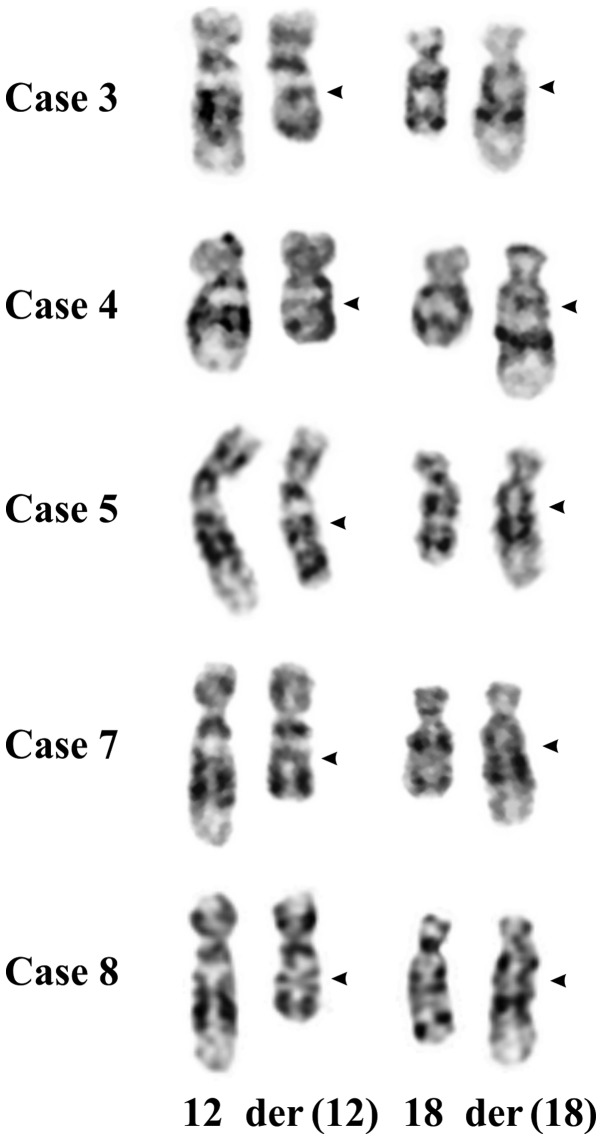
Partial karyotypes of cases 3–5, 7 (lipomas), and 8 (osteochondrolipoma) showing the der(12)t(12;18)(q14~15;q12~21) and der(18)t(12;18) (q14~15;q12~21) together with the corresponding normal chromosome homologs; breakpoint positions are indicated by arrowheads.

In all 12 cases, 8 males and 4 females (Table I), the tumor cells showed cytogenetic recombination between chromosome bands 12q14~15 and 18q12~21 (this was how the cases were selected). Nine lipomas and the osteochondrolipoma (case 8) had a reciprocal t(12;18)(q14~15;q12~21) as the sole karyotypic aberration, one lipoma carried a three-way translocation, t(2;18;12)(q37;q12~21;q14~15), and another tumor had a t(8;9) (p21;q22) in addition to t(12;18). Partial karyotypes of the t(12;18)(q14~15;q12~21) are shown in Fig. 2.

### Real-time PCR

Real-time PCR was performed with two commercially available TaqMan assays for the *HMGA2* gene, one assay for exons 1–2 and the other for exons 4–5, in order to find out whether *HMGA2* was rearranged. Because most *HMGA2* rearrangements take place in intron 3 of the gene, the result is an overexpression of exons 1–3 of *HMGA2*. Thus, the mean quantification cycle (Cq) was compared between the two assays (Table I). Similar Cq values for both assays indicate that both 5′-end (exons 1–2) and 3′-end (exons 4–5) are equally expressed and the *HMGA2* locus is most probably not rearranged. A difference in the Cq values between assays for exons 1–2 and exons 4–5, with the former having a lower Cq than the latter, indicates that 5′-end and 3′-end *HMGA2* exons are unequally expressed and that *HMGA2* is most probably rearranged. In three lipomas (cases 1, 2 and 6), similar Cq values between assays for exons 1–2 and exons 4–5 were found (Table I). In four lipomas and in the osteochondrolipoma (cases 3–5, 7 and 8), on the other hand, the Cq values for exons 4–5 were significantly lower than those for exons 1–2, indicating rearrangement of *HMGA2* (Table I).

### 3′-RACE

**Figure 3:**
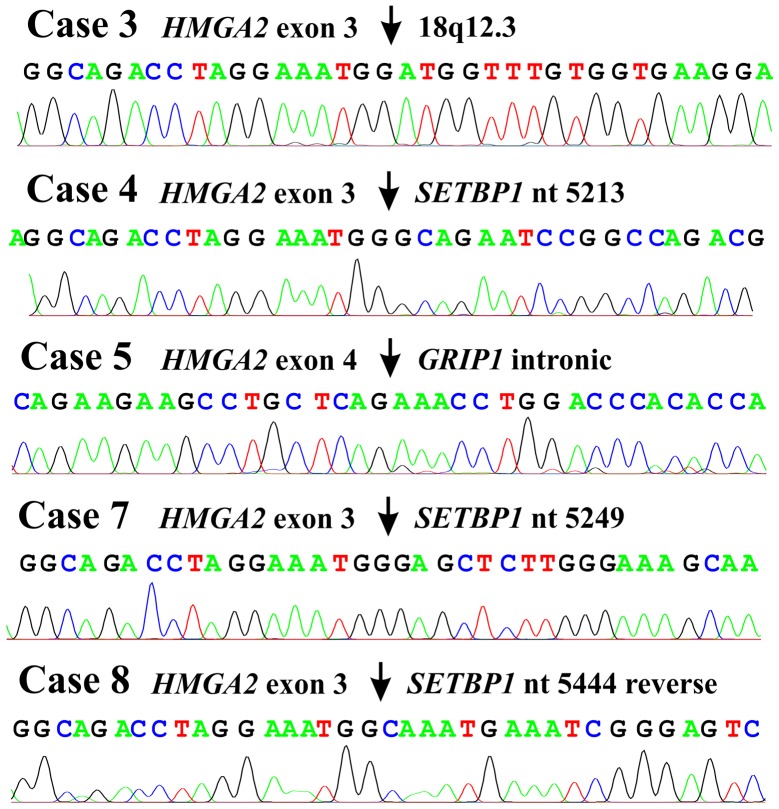
Partial sequence chromatogram of 3′-RACE amplified cDNA fragment showing (arrow) the fusions of *HMGA2*.

3′-RACE on lipomas (cases 3–5 and 7) and the osteochondrolipoma (case 8) (Table I) amplified fragments which by Sanger sequence analysis were found to be chimeric *HMGA2*-cDNA fragments. In lipomas 3, 4 and 7 as well as the osteochondrolipoma, exon 3 of *HMGA2* was fused to sequences from 18q12.3 (Fig. 3) which in lipomas 4 and 7 and the osteochondrolipoma consisted of the 3′-untranslated region of the *SETBP1* gene. In lipoma 3, exon 3 of *HMGA2* was fused with a sequence 10 kbp downstream of the *SETBP1* gene (Fig. 3). In lipoma 5, exon 4 of *HMGA2* was fused to a sequence 500 Mbp distal to *HMGA2* in an intron of *GRIP1* in 12q14.3 (Fig. 3).

**Figure 4:**
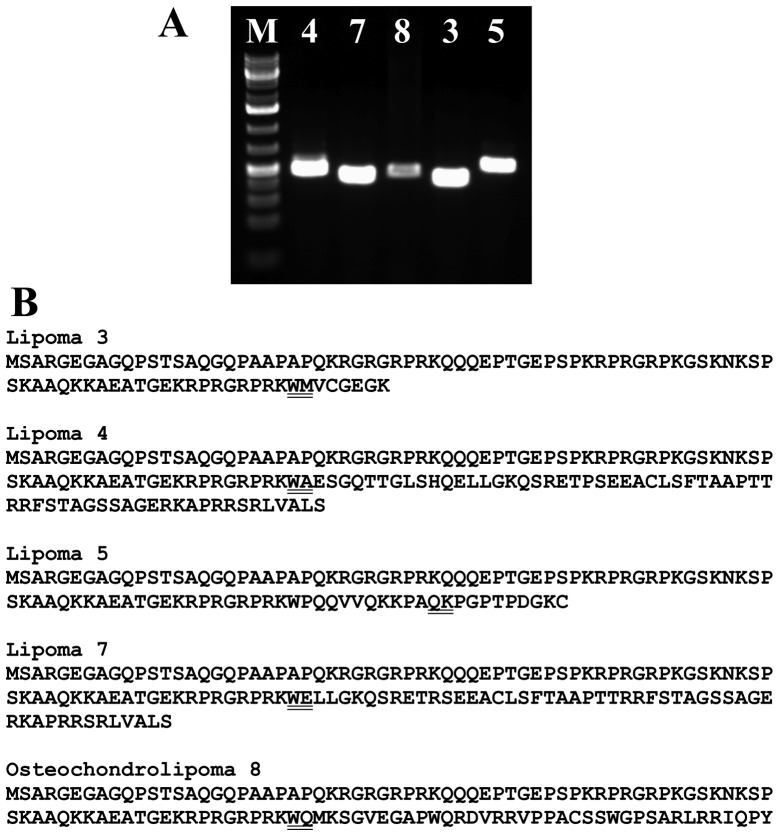
(A) RT-PCR results for the expression of *HMGA2*-fusion in lipomas (cases 3–5 and 7) and osteochondrolipoma (case 8). PCRs were run using the forward primer HMGA2-846F1 and the reverse primer SETBP1-5390R1 for lipomas 4 and 7, primers HMGA2-846F1 and 18q21-Rev1 for case 3, primers HMGA2-846F1 and SETBP1-5325F1 for the osteochondrolipoma, and primers HMGA2-846F1 and GRIP-intrR1 for the lipoma of case 5. M, 1 kbp DNA ladder (GeneRuler; Fermentas, Vilnius, Lithuania). (B) The putative proteins encoded by the *HMGA2*-fusion transcripts.

PCR with the primer HMGA2-846F1 and specific reverse primers amplified a cDNA fragment (Fig. 4A) which by direct sequencing was shown to display the same fusion point as the 3′-RACE amplified fragment (Fig. 3).

## Discussion

We describe here a new recurrent chromosome translocation, t(12;18)(q14~15;q12~21), in lipomas. According to the Mitelman Database of Chromosome Aberrations and Gene Fusions in Cancer (http://cgap.nci.nih.gov/Chromosomes/Mitelman, Database last updated on February 13, 2015), there are 13 cases with aberration involving breakpoints on the long arm of chromosome 18 but none of them had t(12;18) (q14~15;q12~21). Whether some of these cases could nevertheless be cytogenetically similar to the tumors of our series, is a possibility we cannot test.

Because changes of chromosomal bands 12q13~15 in lipomas are almost always associated with rearrangement and/or activation of *HMGA2* (14), we decided to investigate further the possible involvement of *HMGA2* also in the present cases. The real-time PCR and 3′-RACE experiments showed that *HMGA2* was expressed as a chimeric *HMGA2* transcript in five cases. In four lipomas, exons 1–3 of *HMGA2* were fused to a sequence of *SETBP1* (cases 4, 7 and 8) or an intragenic sequence from 18q12.3 (case 3) 10 kbp distal to *SETBP1*. In one tumor (case 5), the translocation t(12;18) resulted in fusion of exons 1–4 of *HMGA2* with an intronic sequence of *GRIP1* which also maps to chromosome band 12q14.3. The ensuing *HMGA2* fusion transcripts code for putative proteins which contain amino acid residues 1–83 of HMGA2 (accession number NP_003474.1) corresponding to exons 1–3 of the gene and amino acid residues from the fused sequences (cases 3, 4, 7 and 8) or, in the tumor of case 5, 94 amino acid residues are translated from exons 1–4 of *HMGA2* and joined with residues from a fused sequence from chromosome 12 (Fig. 4B). Thus, the pattern is similar to that seen in other rearrangements of *HMGA2* found in lipomas, i.e., disruption of the *HMGA2* locus leaves intact exons 1–3 of the gene which encode the AT-hook domains and separates them from the 3′-terminal part of the gene (15). The biological activity of HMGA2 polypeptides in which the acidic tail has been replaced by a variable number of seemingly random amino acid residues, has not been studied in detail. The present data nevertheless add to the evidence that expression of truncated *HMGA2* is involved in the development of lipomas. Fedele *et al* (16) showed that the expression of a truncated form of HMGA2 protein carrying only the three DNA-binding domains, or the expression of a fusion protein carrying the three DNA-binding domains of HMGA2 and the LIM domains of the lipoma preferred partner gene (LPP) protein, caused transformation of NIH3T3 cells. The acquisition of LPP ectopic sequences did not increase the transforming ability of the truncated form of HMGA2 (16). Moreover, transgenic mice expressing a truncated form of the HMGA2 protein develop obesity and an abnormally high prevalence of lipomas (17,18).

The real-time PCR results indicated that full length *HMGA2* transcript was expressed in lipomas 1, 2 and 6 (Table I). In addition, FISH showed that the breakpoint was distal to the *HMGA2* locus in lipomas 1 and 2 (data not shown). Similar data have been reported for lipomas carrying the t(5;12)(q32~33;q14~15) (9,19) where FISH studies (9) have shown that the genomic breakpoints usually lie outside the *HMGA2* locus. Subsequently, Bartuma *et al* (19) showed that 4 out of 5 examined lipomas with t(5;12) had aberrant expression of the entire *HMGA2* gene. Similar findings have also been reported for uterine leiomyomas with rearrangements involving chromosome band 12q15 (20): A study of 38 uterine leiomyomas showed that dysregulation of *HMGA2* expression, not the formation of *HMGA2* fusion transcripts, was the principal pathobiological mechanism in these tumors.

The recurrent *HMGA2* partner gene, *SETBP1*, codes for a protein which contains six regions rich in proline (P), glutamine (E), serine (S), and threonine (T) residues (PEST sequences), three nuclear localization signals, three sequential proline-rich repeats PPLPPPPP at the carboxyl-terminal end, a region homologous to the SKI oncoprotein, and a SET binding region (21). The encoded protein has been shown to interact specifically with the SET protein both in a yeast two-hybrid system and in human cells (21). Constitutional mutations in this gene are associated with Schinzel-Giedion midface retraction syndrome (22). *SETBP1* is otherwise involved in hematologic malignancies (23–25). The oncogenic function of *SETBP1* was first reported in 2006 when a *NUP98-SETBP1* fusion gene was identified in T cell acute lymphoblastic leukemia carrying a t(11;18)(p15;q12) (24). In 2010, *SETBP1* was found to be overexpressed in a case of AML cytogenetically characterized by a t(12;18)(p13;q12) targeting *ETV6* in the second breakpoint (23). The same study also showed that *SETBP1* overexpression is a recurrent molecular event in AML (found in 53 of 192 patients) and is associated with shorter overall survival, especially in elderly patients (23). Recently, recurrent mutations in *SETBP1*, frequently targeting the SKI-homologous domain, have been identified in several types of myeloid malignancies, including chronic and acute myeloid leukemias (25 and refs. therein). In the three *HMGA2-SETBP1* fusions described here, the breakpoint in *SETBP1* occurred in the 3′-untranslated region (3′-UTR). This 3′-UTR is 4.8 kbp long, or almost half the size of the 9.9 kbp long transcript mRNA of *SETBP1* (sequence with the accession number NM_015559 version 2). The function of this 3′-UTR is unknown but presumably it contains sequences that influence the expression of *SETBP1* (26).

Case 8, which also had a *HMGA2-SETBP1* fusion, was diagnosed as osteochondrolipoma (Fig. 1). This is a very rare type of tumor on which no prior cytogenetic or molecular genetic information exists. A search in ‘PubMed' using the term ‘osteochondrolipoma' yielded 6 articles (27–32). As the name indicates, the tumor is characterized by the presence of mature fatty tissue together with cartilage and bone formation with lipocytes, chondrocytes, and osteoblasts being the predominant cell types (31). The pathogenesis of osteochondrolipomas is unknown. Lin *et al* (33) reported that mesenchymal stem cells (MSCs) may be found in human lipomas and that they have characteristics similar to those of MSCs derived from adipose tissue. Thus, lipoma-derived MSCs can differentiate into adipocytes, osteoblasts, and chondrocytes after induction (33) and could be the source of the different cell types in these tumors. The present case with a t(12;18)(q14~15;q12~21) and an *HMGA2-SETBP1* fusion identical to those found in the ordinary lipomas further supports the association both pathogenetically and otherwise between osteochondrolipomas and other lipoma subtypes. Worthy of mention is that the reciprocal translocation t(3;12)(q27~28;q13~15), i.e., the most common translocation in lipomas, has also been observed in three cases of osteolipoma (34). Evidently, the special phenotypes of these lipoma variants cannot be attributed to the tumor pathogenetic mechanism.

The present study provides yet another example of the fact that what at the cytogenetic level appears to be similar is in fact heterogeneous at the molecular level (9,19). This may reflect the intriguing pathogenetic role of *HMGA2*, which seems to be entirely different from the highly specific gene fusions present in, for example, myxoid liposarcomas (14).

## Figures and Tables

**Table I:** Clinical, cytogenetic and molecular data on the 12 benign fat cell tumors.

Case no.	Gender/ age (years)	Diagnosis	Location	Karyotype	Cq, Ex1-Ex2 of *HMGA2*	Cq, Ex4-Ex5 of *HMGA2*	Cq of *ACTB*	*HMGA2* fusion
1	M/58	Lipoma	Intramuscular, left thigh	46,XY,t(12;18)(q14~q15;q12~q21)[12]/46,XY[3]	30.97	28.23	23.41	Not done
2	F/44	Lipoma	Intramuscular, right elbow	46,XX,t(12;18)(q14~q15;q12~q21)[16]	29.76	27.58	25.27	Not done
3	M/54	Lipoma	Intramuscular, right deltoid	46,XY,t(12;18)(q14~q15;q12~q21)[12]/46,XY[3]	**28.58**	**36.84**	24.33	*HMGA2*-sequence from 18q12.3
4	F/34	Lipoma	Intramuscular, right deltoid	46,XX,t(12;18)(q14~q15;q12~q21)[14]	**30.10**	**39.69**	24.33	*HMGA2-SETBP1*
5	M/38	Lipoma	Left thoracic wall	46,XY,t(12;18)(q14~q15;q12~q21)[15]	**28.73**	**38.84**	22.45	*HMGA2-GRIP1*
6	F/28	Lipoma	Intramuscular, left thigh	46,XX,t(12;18)(q14~q15;q12~q21)[14]/46,XX[1]	32.57	30	22.96	Not done
7	F/61	Lipoma	Intramuscular, right splenius capitis muscle	46,XX,t(12;18)(q14~q15;q12~q21)[12]/46,XX[3]	**26.28**	**39.36**	23.67	*HMGA2-SETBP1*
8	M/55	Osteochon-drolipoma	Intramuscular, subscapularis muscle	46,XY,t(12;18)(q14~q15;q12~q21)[15]	**30.04**	**35.64**	24.97	*HMGA2-SETBP1*
9	M/55	Lipoma	Intramuscular, right infraspinatus muscle	46,XY,t(2;18;12)(q37;q12~q21;q14~15)[9]	Not done	Not done	Not done	Not done
10	M/15	Lipoma	Foot, right intrametatarsal	46,XY,t(12;18)(q14~q15;q12~q21)[15]	Not done	Not done	Not done	Not done
11	M/64	Lipoma	Right groin	46,XY,t(8;9)(p21;q22),t(12;18)(q14~q15;q12~q21)[10]	Not done	Not done	Not done	Not done
12	M/56	Lipoma	Intramuscular, left deltoid	46,XY,t(12;18)(q14~q15;q12~q21)[5]/46,XY[5]	Not done	Not done	Not done	Not done
